# Large-scale structure formation in ionic solution and its role in electrolysis and conductivity

**DOI:** 10.1371/journal.pone.0213697

**Published:** 2019-03-26

**Authors:** Chut-Ngeow Yee, C. H. Raymond Ooi, Luck-Pheng Tan, Misni Misran, Nyiak-Tao Tang

**Affiliations:** 1 Department of Physics, Faculty of Science, University of Malaya, Kuala Lumpur, Malaysia; 2 Prime Oleochemicals Industries Sdn. Bhd., Petaling Jaya, Malaysia; 3 Department of Chemistry, Faculty of Science, University of Malaya, Kuala Lumpur, Malaysia; US Naval Research Laboratory, UNITED STATES

## Abstract

That water may not be an inert medium was indicated by the presence at water’s interfaces a negatively charged solute free zone of several hundred microns in thickness called the exclusion zone (EZ). Further evidence was demonstrated by Ovchinnikova’s experiments (2009) showing that water can store and release substantial amount of charge. We demonstrate that the charge storage capacity of water arises from highly stable large-scale ionic structures with measurable charge imbalances and discrete levels of charge density. We also show evidence that the charge zones formation requires ionic solutes, and their formation correlate to large change in conductivity, by as much as 250%. Our experiments indicate that large-scale structuring plays a pivotal role in electrolysis and conductivity in ionic solution. We propose that water is an electrochemically active medium and present a new model of electrolysis and conductivity in ionic solution.

## Introduction

The presence of solute-free zones, called the exclusion zones (EZ) near water’s interfaces is a phenomenon that is being extensively studied experimentally and theoretically in recent years [1] [2] [3] [4] [5] [6] [7]. This solute-free zone typically spans several hundred microns and is observed in a variety of hydrophilic surfaces; including natural and artificial hydrogels, the water-air interface, biological surfaces, hydrophilic polymers and metallic surfaces. Electrical measurements show large negative potential within the zone, of the order of 100 mV, and pH measurement show a large concentration of H^+^ beyond the zone [2]. EZ has also shown to absorb radiant energy to build and maintain its structure, with peak absorption at the 270 nm wavelength [8]. NMR spectroscopy shows that it is highly restricted and dynamically more stable [9]. It can also exert mechanical forces measurable using a laser tweezers system [10]. Refractive index measurement show a 10% increase in the EZ [11]. Cryogenic scanning electron microscopy suggests that EZ consists of high density water with a cell-like wall structure [12].

The recent investigations lend strong evidence that the EZ is a coherent large-scale structure. The large extent and substantial concentration of negative potential in EZ is particularly surprising. It is indicating that water is not a neutral and inert medium in electrochemical processes. The electrically active nature of water is further demonstrated by Ovchinnikova et al. [13] who use an electrolysis setup to investigate the charge storage characteristic of water. They demonstrated with the aid of pH sensitive dye that an alkaline and an acidic zone form at the cathode and the anode respectively. The two zones are charge bearing and significant amount of discharge, as much as 70% of the input charges can be obtained from the charged zones. This provides evidence that the water is not just electrically active at the interface EZ, but also in the bulk water beyond the interfaces.

In this paper we extend Ovchinnikova experiments to further investigate the nature of the charge zones formation. Our experimental setup consists of a long column of water with electrodes at both ends and a series of equally spaced probes along the length to measure the voltages at each point. With this setup we are able to ascertain that zones with measurable potential differences–to as much as 2.5 V form in the water. The zones were highly stable and show discrete voltage levels. The zones resist mutual neutralization despite being drawn in close proximity to each other by electrostatic attraction, and they can maintain their voltage levels for well over 10 hours. We also show evidence that the charge zones buildup bear strong correlation to significant change in conductivity–by as much as 250%.

Our investigations support the growing evidences that water is an electrochemically active medium. We conjecture that this is due to water’s capacity to form large-scale structures that is able to retain excess charges. We put forward the hypothesis that the charge zones are coherent colloid-like structures formed by water dipoles with ionic solutes and electrochemical by-products and investigate the relationship between ionic solute, charge zone creation and conductivity. We conclude this paper by proposing a new model of electrolysis and electrical conductivity in ionic solutions and explore the role of water as an active agent in electrochemical reactions.

## Experimental methods

### Electrical control system

**Fig 1:**
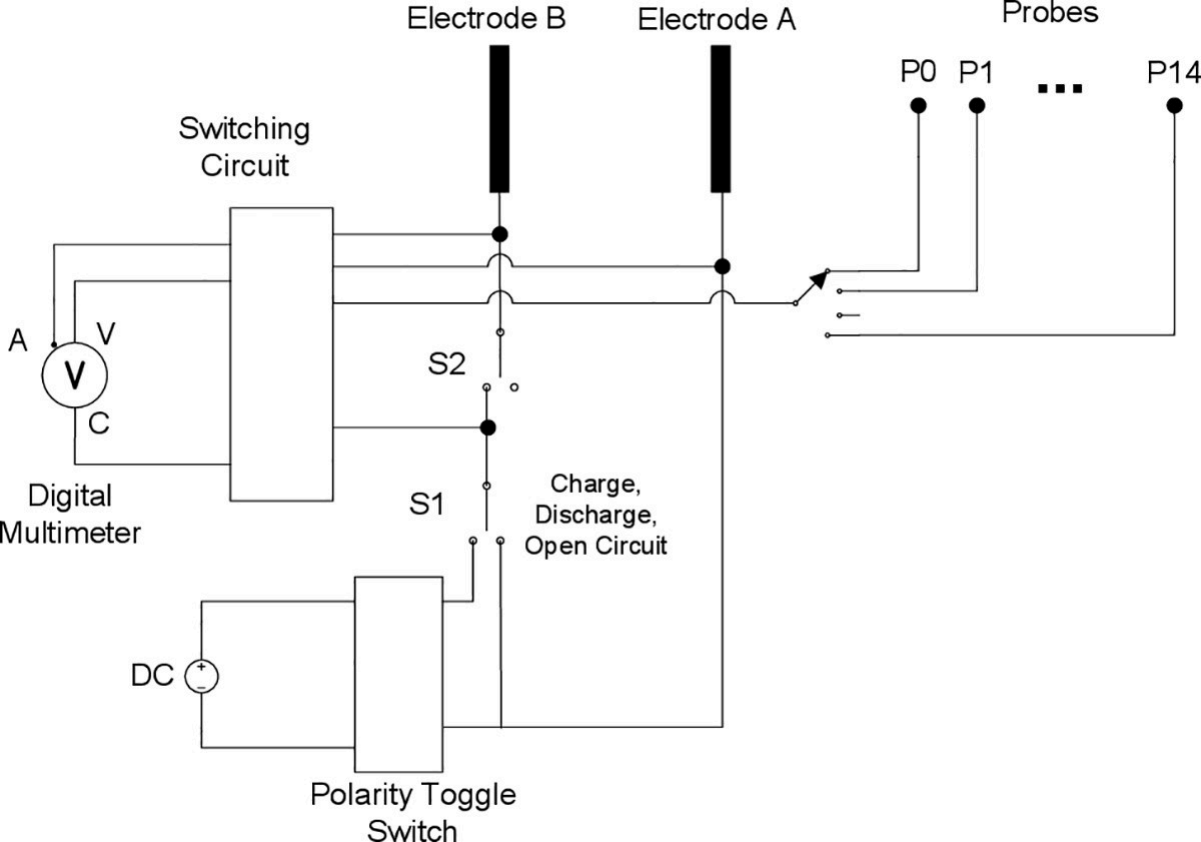
Control system schematic diagram. The test station is a simple electrolysis setup with computer controlled circuitry to connect to a digital multimeter for current and voltage measurements. The circuit supports voltage measurement of up to 15 probes against electrode A or B.

The test station used in our experiments is a computer controlled electrolysis setup with two electrodes and a DC power source. The schematic diagram of the control system is shown in Fig 1. Switch S1 is a three poles switch that allows switching the circuit to charge, open circuit, or discharge mode. In Charge mode the electrodes are connected to the power source, the Open Circuit mode will break the circuit between the electrodes and the Discharge mode will short circuit the electrodes to discharge the stored charges. The polarity inverse switch is to control the polarity of the voltage applied to the electrodes, so the Charge mode is further divided into Forward Charging and Reverse Charging. The switching circuit supports up to 15 probes that can be placed anywhere in the water medium and voltages can be measured relative to electrode A or B.

The voltage and current are measured with a programmable Rigol DM3068 6½ digits Digital Multimeter (DMM). Switch S2 is to momentarily break the circuit and route the current into the DMM for current measurement. The DC power source is a generic brand adjustable 0-60V/0-3A power supply, with a measured ±60 mV fluctuation within our range of operation. The experiment is equipped with a PT100 sensor to track temperature variations. Temperature reading is tagged to every data point.

The control system is constructed using USB relay modules. A program written in C#.NET controls the switches and logs the data into a database. Measurements are typically carried out once per minute. It takes about 2.5 seconds to switch through the 15 probes.

### Electrochemical setup

**Fig 2:**
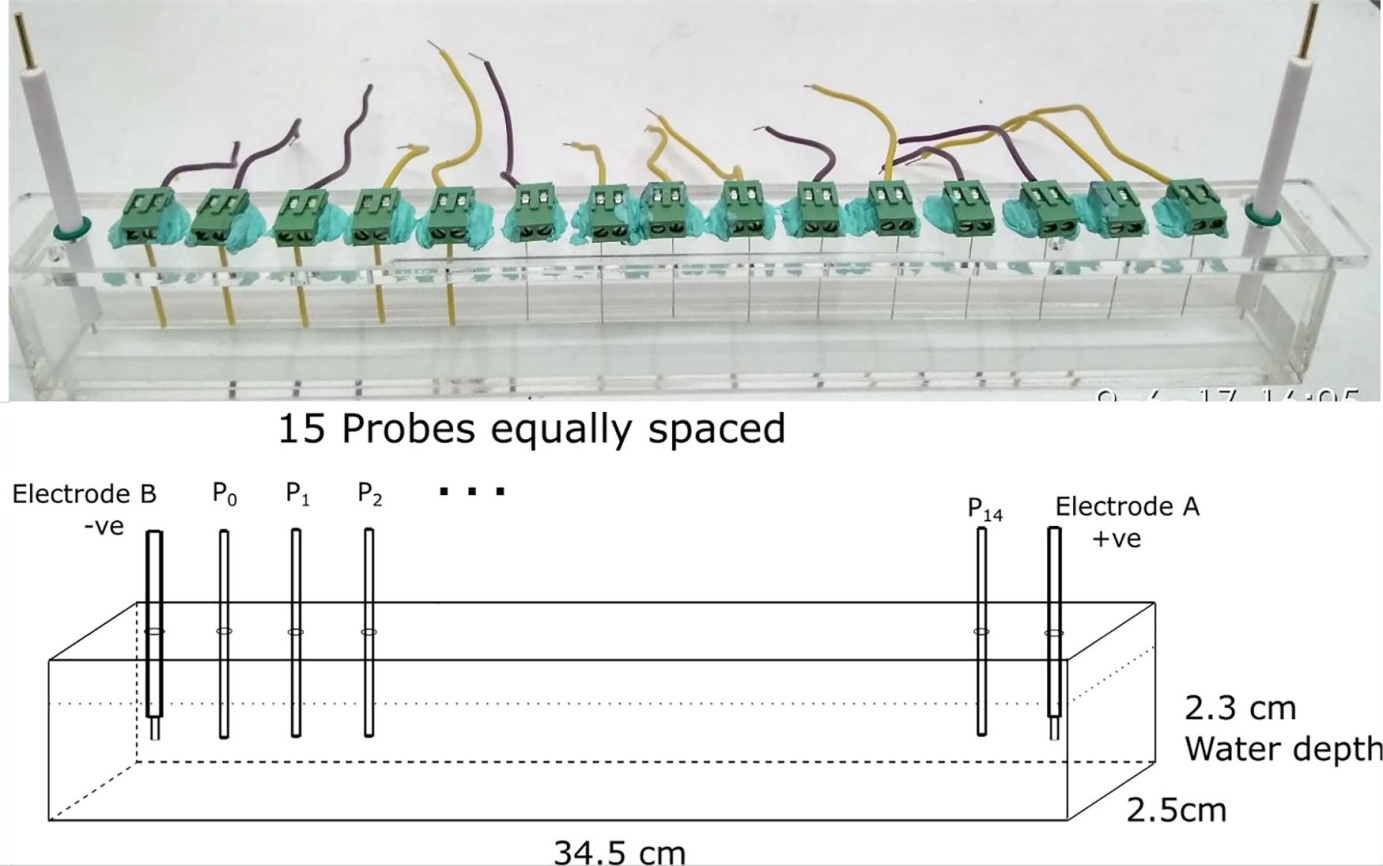
The electrochemical setup. The electrochemical setup consists of an acrylic container of base dimension 34.5 cm x 2.5 cm. 200 ml of solution is used, which gives a liquid depth of 2.3 cm. 15 probes are placed at equidistance between the electrodes. The electrodes are platinum rods of 1 mm diameter, 5 mm length. The probes are made of shielded platinum wires placed at a depth of 1.4 cm, or 0.9 cm from the bottom.

The electrochemical setup, as shown in Fig 2, consists of a long acrylic container of base dimension 34.5 cm × 2.5 cm. We typically use 200 ml liquid volume, which gives a water depth of 23 cm. Two platinum rod electrodes of 1 mm diameter × 5 mm length are positioned at both ends of the container. Fifteen probes are placed at equidistance between the electrodes. The probes are 26 gauge platinum wire shielded with PVC insulation with only the lower tip of the wire in contact with the solution. They are placed 2.1 cm apart, submerged at a depth of 1.4 cm (or 0.9 cm from bottom).

## Probing charge zones formation

**Fig 3:**
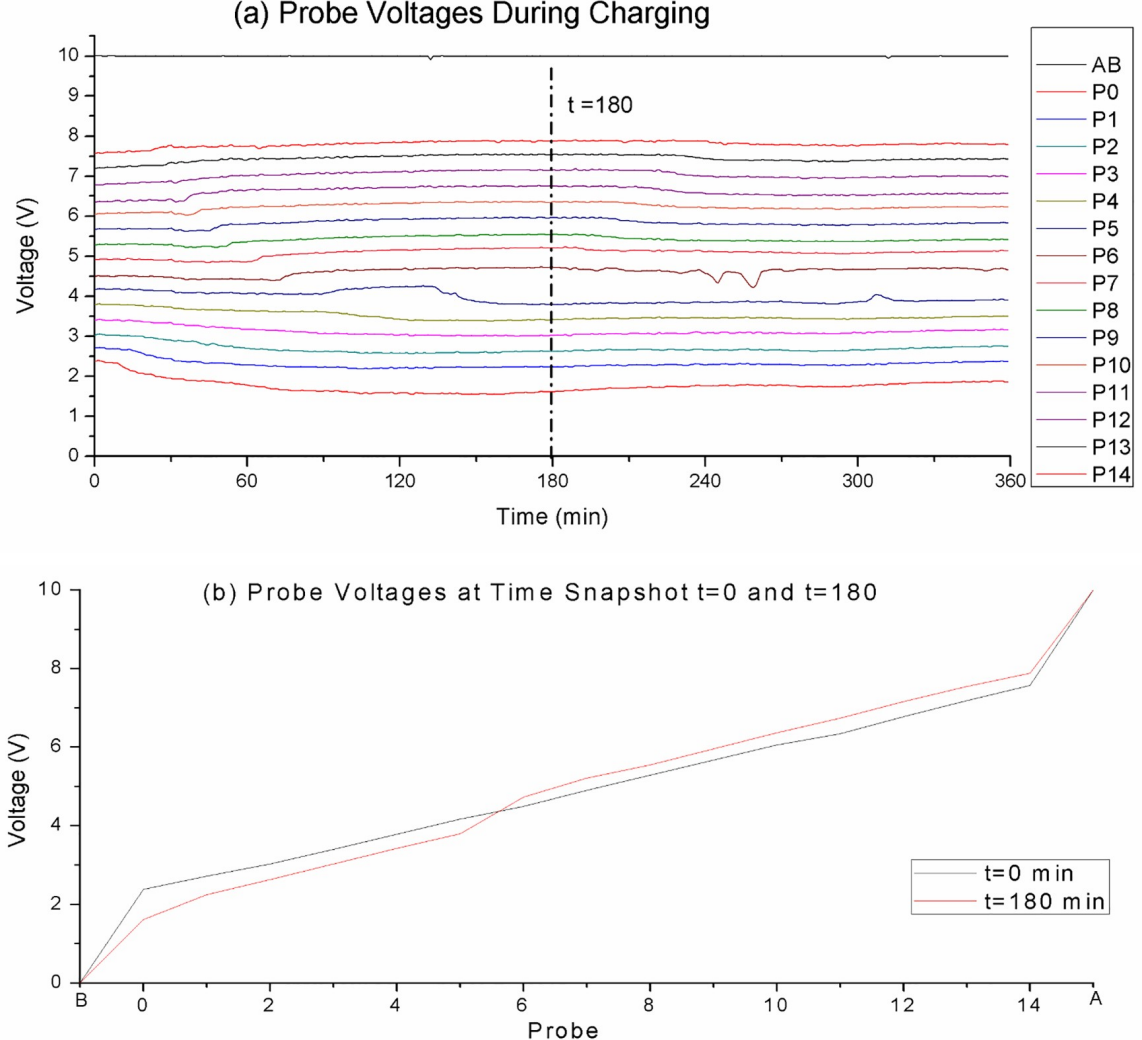
Probes voltages during charging of 20 mM Na_2_SO_4_ solution over 6 hours at 10 V. (a) The probe voltages vs. time graphs. We can see that the top 9 probes drifting upwards, while the bottom 6 probes drifting downwards leaving a relatively large voltage gap between probes P_5_ and P_6_. (b) The probe voltage profiles at time snapshots t = 0 and t = 300 min. We can see that at t = 180 probes P_0_..P_5_ have acquired negative biases, while probes P_6_..P_14_ acquired positive biases and there is a clear crossover point between P_5_ and P_6_.

Fig 3 shows probe voltages vs. time graphs of charging 20 mM Na_2_SO_4_ solution over 6 hours at 10 V. We can see the upwards trend of the top 9 probes over time, while the bottom 6 probes are showing a downward trend, leaving a relatively large voltage gap between probes P_5_ and P_6_.

Fig 3(B) shows the probe voltage profiles at time snapshots t = 0 and t = 180 min. We can see that at t = 180 probes P_0_..P_5_ have acquired negative biases, while probes P_6_..P_14_ acquired positive biases and there is a crossover point between P_5_ and P_6._

**Fig 4:**
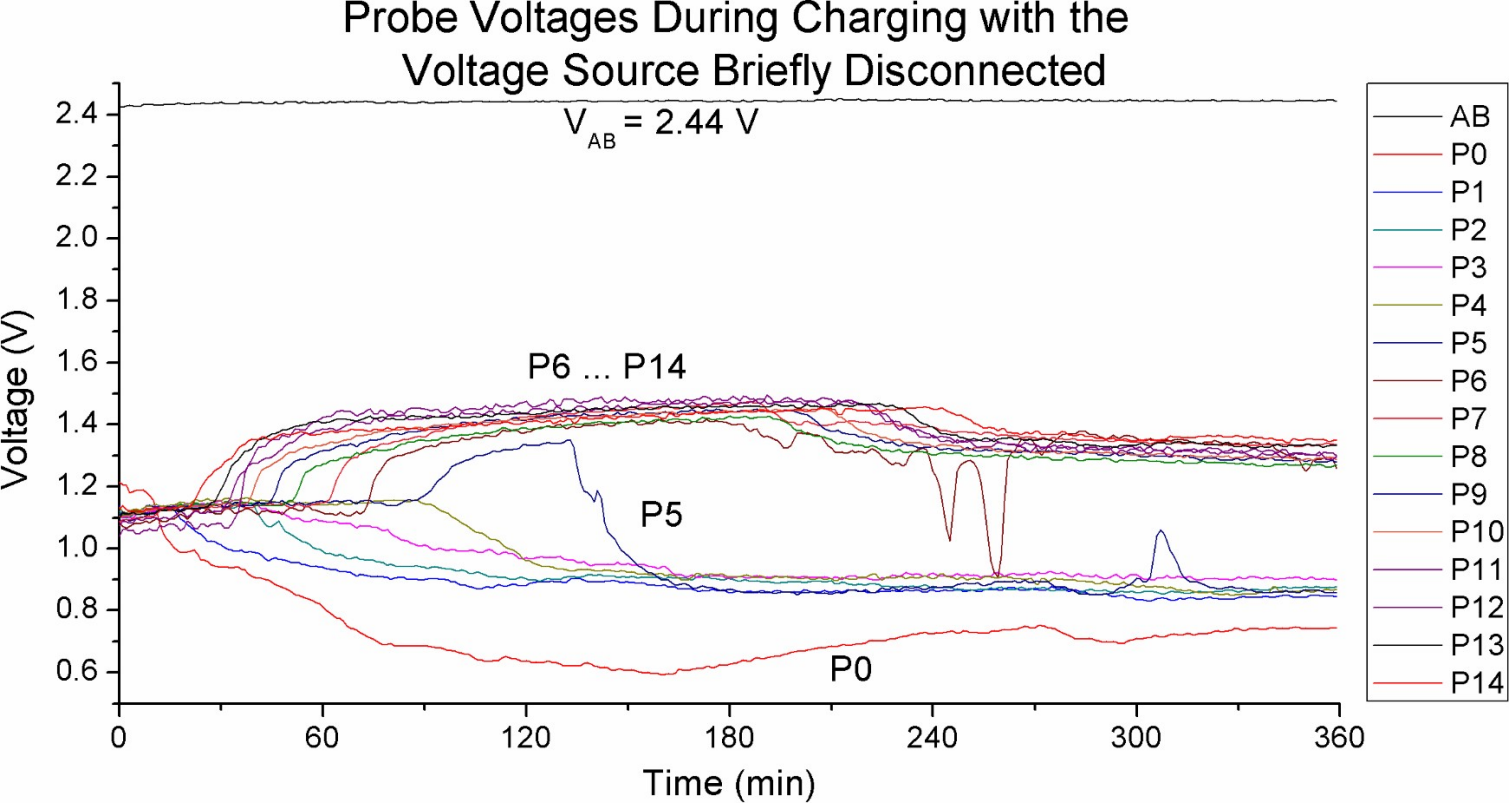
Probe voltages with power supply temporarily disconnected. We can see the probes separating into two zones that are approximately 0.6 V apart.

Fig 3(B) suggests that there is local charge build up in the solution and the voltage biases appear to be constant. But the comparison is between two time snapshot that are 3 hours apart, so what is the absolute voltage at each probe position? We do not have a zero reference point to do absolute measurements, so we interrupted the charging briefly (for about 2.5 sec) so that we can measure the probe voltages without the external power source. Fig 4 plots the voltage vs time graph for the probes. Measurements were with respect to the negative electrode. Measurements for Figs 3 and 4 were done concurrently in interleave fashion.

From Fig 4 we can see that the probes are indeed splitting into two distinct zones of approximately 0.6 V apart. Our result confirms Ovchinnikova’s observation that positively (acidic) and negatively (alkaline) charged zones form in the water. We can also see the progression of the charge zone built up. As expected the charged zones start forming from the electrodes, so we see P_0_ swing to the negative side, followed by P_1_ etc. Similarly on the positive side the charge zone build from P_14_ followed by P_13_ etc. The time scale is rather slow, it took 120 min for the zones to meet in the middle. The boundary point is between P_5_ and P_6_. We can see P_5_ drifted up to the positive side first before dropping down and settle on the negative side.

The voltage between the electrodes V_AB_ is 2.44 V (Fig 4). This is the maximum voltage that we observed in Na_2_SO_4_ solution. We have tested with charging the solution up to 90 V but once the external voltage source is removed, V_AB_ will decay to the 2.44 V level within milliseconds. Here V_AB_ appears constant but when the charging voltage and/or ion concentration is low it could take considerable length of time, of the order of hours, for this voltage to slowly build up to the threshold level. V_AB_ is unstable when the solute concentration is extremely low (lower than 1 mM for H_2_SO_4_).

In Fig 4 at t = 180 min onwards we see all the positive sides probes drop by the same quanta of approximately 0.2 V starting from P_6_ in the middle spreading outwards in sequence to P_14_. This is a significant event that we will elaborate later.

V_AB_ is much higher than the 0.6 V difference between the positive and negative charge regions. This means there is a region of much higher charge concentration near the electrodes. This will become apparent in later diagrams.

## Some general characteristics of charge zones formation

We have performed over 200 experiments on various solutes over a wide range of ion concentration, from deionized water to 320 mM solutions. We observed stable large-scale charge zone formation on all our experiments when the ion concentration is above 1 mM. But even with deionized water where large-scale charge zone is not observed, we have evidence of the formation and rapid migration of small charged zone fragments. We will dedicate a section on the surprising and revealing behaviour of deionized water later. We can conclude with high certainty that charge zone formation is inseparable from electrical conductivity in ionic solution.

The general characteristic of the charge zones is that they will form from the end of the container and grow towards the middle. This particular formation pattern is governed by the long narrow tube physical constrain of the container. With containers that allow higher degree of freedom, we have seen much more complex charge zones formation and movement patterns.

The shape and surface area of the electrodes affect the charging current and the rate of charge zone formation. This is a minor factor within the context of the investigations presented in this paper. We generally adjust the charging voltage to limit the charging current to below 10 mA. This is the region that show the most orderly charge zone formation, and where the charge zones assert the greatest effect on the charging current. High charging current will cause high rate of bubbles formation, which can induce disruptive turbulences near the electrodes.

**Fig 5:**
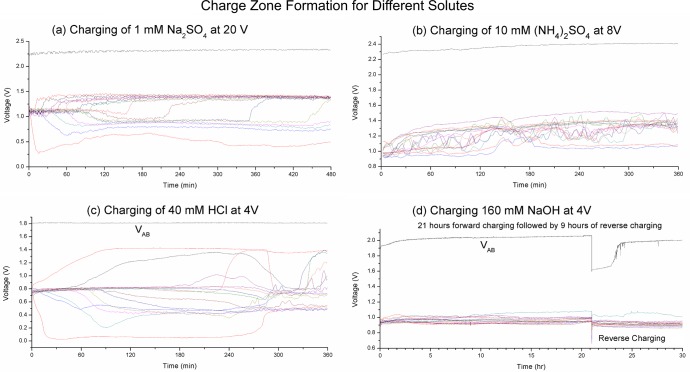
Charge zones profile for various solutes. (a) Charging of 1 mM H_2_SO_4_ at 20 V. (b) Charging of 10 mM (NH_4_)_2_SO_4_ at 8V. (c) Charging of 40 mM HCl at 4V. (d) Charging of 160 mM NaOH at 4V.

The relative size of the positive and negative charge zones is another interesting characteristic of charge zones formation, and the meeting point of the two zones can be highly unbalanced. This particular aspect is primarily determined by the type of solvent and is minimally affected by ion concentration and other factors. We conjecture that the relative size of charge zones is related to the electrolytic strength of the ions involved in the formation of the respective zones–positive ions corresponds to the negative zone, and negative ions to the positive zone. Higher electrolytic strength of the positive ions will give rise to larger negative zone, and vice versa. We will elaborate further about this in Fig 5B and Fig 5C below.

Fig 5 presents some representative results from our experiments. These are graphs of probe voltages during charging with the voltage source temporarily disconnected.

Fig 5A show the charge zone for 1 mM Na_2_SO_4_ charging at 20V. This is near the lowest ion concentration required for large-scale stable charge zone to form. The shape of the charge zone is similar to that shown in Fig 4 for 20 mM Na_2_SO_4_, but the lines are jagged, indicating instabilities. Indeed the charge zone collapsed when the charging voltage is reversed (not shown in graph).

Fig 5B show the charging of 10 mM (NH_4_)_2_SO_4_ at 10 V. This is a weaker electrolyte than H_2_SO_4_, and we see here the charge zone is less extensive and less defined. The general trend is that stronger electrolyte strength of the solute will produce more extensive and defined charge zones. We see 13 probes swing up to the positive side and 2 probes swing down to the negative side, indicating a 13:2 ratio in the relative size between the positive and the negative zones. The SO_4_^2-^ ions migrate to the positive electrode and are involved in the formation of the positive zone, while the NH_4_^+^ ions migrate to the negative electrode and are involved in the formation of the negative zone. We conjecture that the large imbalance in the charge zone size is related to the differences in electrolytic strength of the ions involved. Here the SO_4_^2-^ ion has a stronger electrolytic strength than the NH_4_^+^ ion, and thus we see a much larger positive zone.

Fig 5C shows the charging of 40 mM HCl at 4V. Here we see 2 probes drifted up to the positive side while 13 probes drifted down to the positive side. As with Fig 5B we conjecture that the highly imbalanced relative size of the charge zones is due to the H^+^ ions having a higher electrolytic strength than the Cl^-^ ions.

Fig 5D show the charging of 160 mM NaOH at 4V. We do not see any charge zone formed at any of the probe positions even after 21 hours of charging. However the initial 0.5V dip in V_AB_ at reverse cycle followed by a sudden jump indicates that charge zone did form near the electrodes. The charge zones have very substantial charge density–it took more than 3 hours to dissipate them at the reverse cycle.

The V_AB_ value represents the maximum voltage that can be sustained by the charge zones. This is another important characteristic of charge zone formation that is primarily determined by the type of solutes and it is independent of the charging voltage. We have seen V_AB_ ranging from 1.8–2.5 V with different solutes. V_AB_ is always lower than the charging voltage. When the external voltage source is disconnected we will see an initial sharp decay followed by a much slower rate of dissipation. We typically configure a 50–100 ms delay before taking measurement to avoid the initial sharp decay region.

Table 1 tracks the V_AB_ value of 20 mM Na_2_SO_4_ at different charging voltage. Each experiment was run for 27 minutes and measurements were taken once per minute. We allowed the voltage to stabilize for 10 minutes, and use the last 18 measurements in the calculation. The table shows that when the charging voltage is ≥ 4V V_AB_ converges to a threshold value near 2.43 V. This voltage is very stable and it is independent of the charging voltage.

**Table 1:** V_AB_ value of 20 mM Na_2_SO_4_ at various charging voltages.

Charging Voltage (V)	2	3	4	6	8	12	16	20
V_AB_ Average (V)	1.871	2.336	2.383	2.413	2.417	2.429	2.427	2.429
Standard Deviation	0.003	0.017	0.028	0.009	0.005	0.003	0.003	0.002

Our experiments are carried out in an unair-conditioned room where the air temperature has a daily 26–34°C range. We did not detect any significant effect of temperature variation with our scope of investigations. However, we do expect that charge zones formation will behave differently when the temperature is towards the freezing or the boiling point. This is a subject for further investigations.

The equipment is situated in an isolated corner away from external disturbances. There is no through traffic, machineries or source of vibrations near the experimental setup. However, convection flows do happen naturally due to the electrochemical processes. Gas production at the electrodes will cause convection flow near the electrodes. We have observed that the charge zones have different densities and rates of mobility. At the junction between the positive and negative charge zones usually some pressure will build up, and we will see a sudden reorganization where the lighter charge zone will slide over the denser charge zone. For example in Fig 5C at t = 270 min we see a disruption that may indicate such an event. Such sudden reorganization is a common occurrence that is usually associated with a significant change in conductivity. We will elaborate the strong correlation between charge zone formation and conductivity in a later section.

## The discrete nature of the charge zones

**Fig 6:**
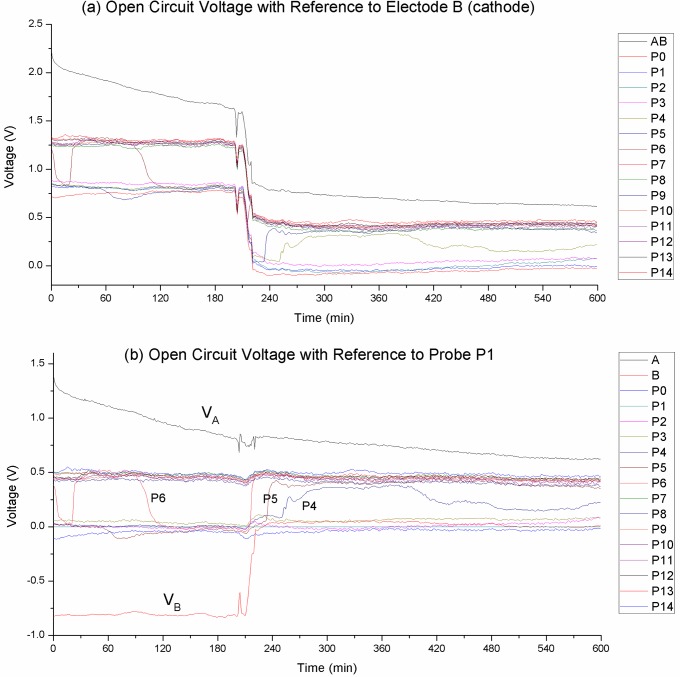
Tracking the probe voltages over 10 hours of open circuit. (a) Probe voltages plotted with reference to electrode B. The abrupt change in the graphs indicates a catastrophic happening at the reference point. (b) The same graphs plotted with P_1_ as the zero reference present a more coherent picture. We can see the probe voltages took on discrete values. P_0_ …P_5_ assume the same voltage at the negative side, while P_6_ …P_14_ assume voltage at the positive side. V_B_ initially assumes a much higher negative voltage, but at t = 200 min the voltage collapse abruptly and drop down to the P_0_…P_5_ level.

Fig 3 and Fig 4 shows the gradual formation of charge zones with an external voltage source. How do the charge zones behave when the voltage source is removed? Fig 6 tracks the probe voltages for 10 hours after 6 hours of charging with a 10 V power source. Measurements were done relative to electrode B (the cathode during charging).

Fig 6(A) plots the data points with electrode B as the reference. The abrupt change in the graphs indicates a catastrophic happening at electrode B making it an unsuitable reference point. Fig 6(B) is the same graphs but plotted with P_1_ as the zero reference. Here we see a much more coherent picture:

- P_0_ …P_5_ assume the same negative voltage, while P_6_ …P_14_ assume the same positive voltage. The two zones are separated by a 0.5 V potential difference.
- The probes maintain the same voltage level through the 10 hours period, with the exception of the probes at the boundary of the two zones, P_4_, P_5_ and P_6_.
- The negative electrode (B) shows a much higher negative voltage level initially. The voltage was stable up to t = 200 min at which point it collapsed abruptly and the voltage dropped to the P_0_…P_4_ level. This gives evidence that the charge zones are large-scale structures that hold fixed or quantized amount of charges. Also take note of the global disturbances during this event. We conjecture that the charge zone collapse released a flood of free electrons into the solution.
- The positive zone shows similar discrete behavior, with P_7_…P_14_ assuming the same stable voltage level. The positive electrode (A) starts high and slowly converges to the stable voltage level occupied by the positive side probes. It did not do a quantum jump like the negative electrode.

**Fig 7:**
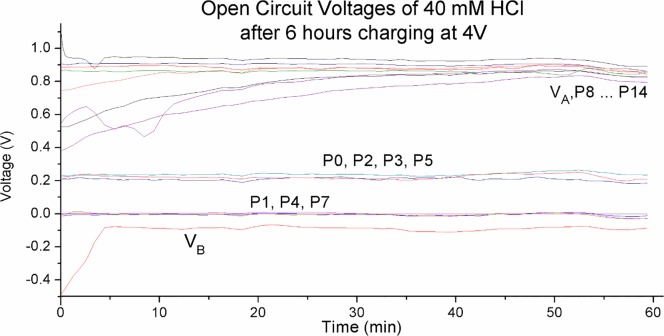
Open circuit probe voltages of 40 mM HCl after 6 hours charging at 4V. The graphs are plotted with reference to probe P_1_. We can see three discrete voltage levels on the negative charge zone.

Fig 7 is another demonstration of the discrete nature of the charge zones. The graphs track the probe voltages of 40 mM HCl after charging at 4 V for 6 hours (refer to Fig 5C). The graphs are plotted with reference to probe P_1_. We can see three discrete voltage levels at the negative zone. Electrode B shows a rapid initial decay then settles on a stable voltage level. At 0.05 V higher we see probes P_1_, P_4_, P_7_ occupying another stable voltage level, and a further 0.2 V higher we see P_0_, P_2_, P_3_, P_5_ occupying yet another level. P_6_ was fluctuating between two different voltage levels, it was omitted for clarity.

The positive charge zone however does not show such strong and obvious discrete nature. We do see voltage levels that can remain stable for hours, but the probes do show varying voltage levels that are converging to the stable voltage level at a very slow rate.

## The relationship between charge zones and conductivity

Gradual but significant change in charging current, by as much as 250% over a span of hours, is a prominent effect we observed in our experiments. In this section we present evidence that such large changes in conductivity is related to charge zone formation in the solution.

**Fig 8:**
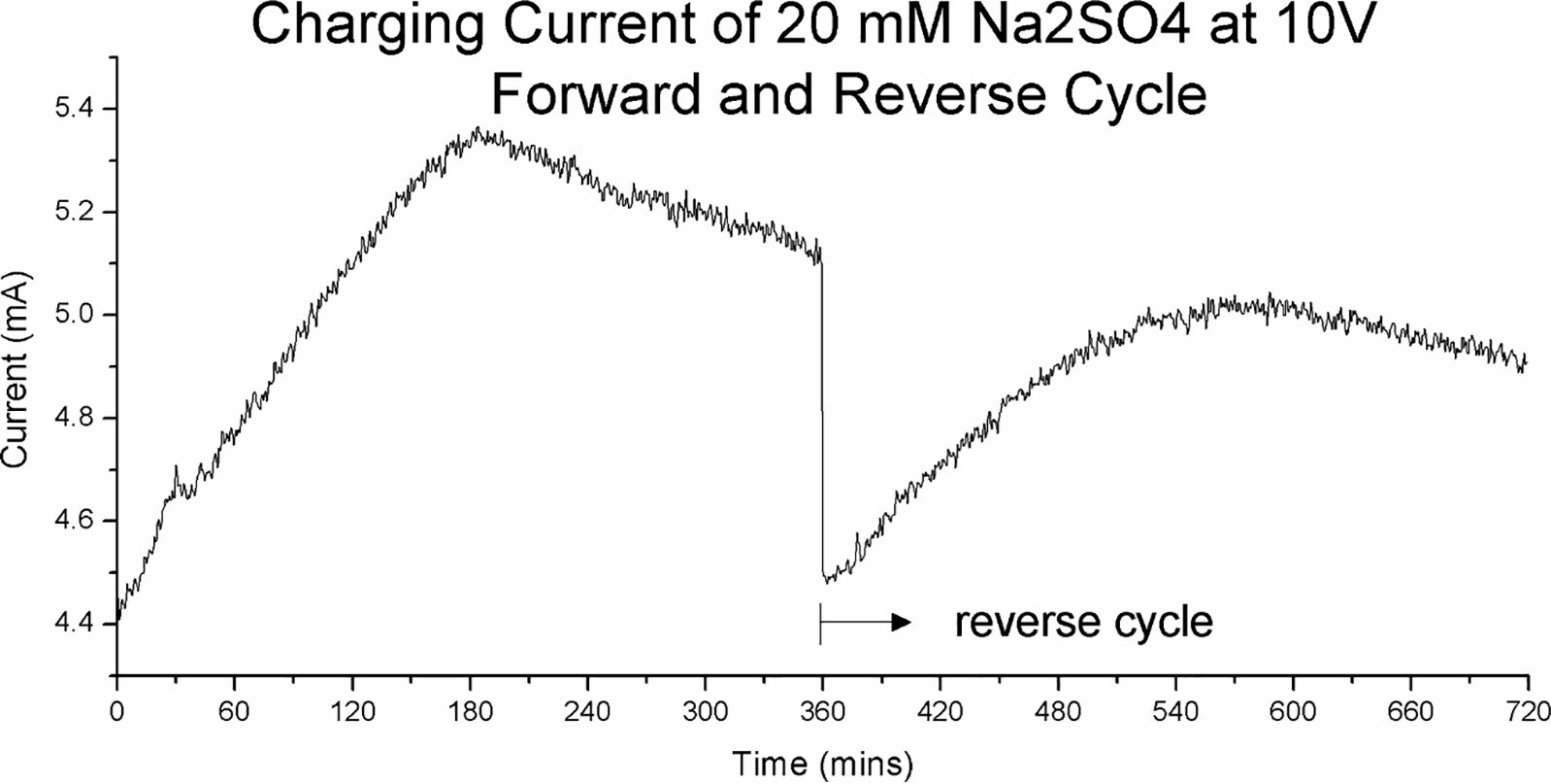
The charging current of 20 mM Na_2_SO_4_ at 10 V over a 6-hour forward cycle followed by 6 hour of reverse cycle. The current rose from 4.5 mA to a peak of 5.4 mA–a very significant 20%—before coming down slowly again at t = 180 min. When the charging direction is reversed the current plunge back down to near the starting level and begin to rise slowly again.

Fig 8 shows the charging current graph of Na_2_SO_4_ at 10 V. It is the current graph for the same experiment presented in Figs 3–6. This figure shows 6 hr of forward charging followed by 6 hr of charging with reverse polarity. Although the forward and reverse charging currents are shown continuously, there is a 10 hr open circuit stage between the two phases.

The current increased from 4.5 mA to a peak of 5.4 mA–a very significant 20%—before coming down slowly again at t = 180 min. When the charging direction is reversed the current plunge back down to near the starting level and begin to rise slowly again. This would rule out the possibility that the current increase was due to built-up of electrolysis by-products.

**Fig 9:**
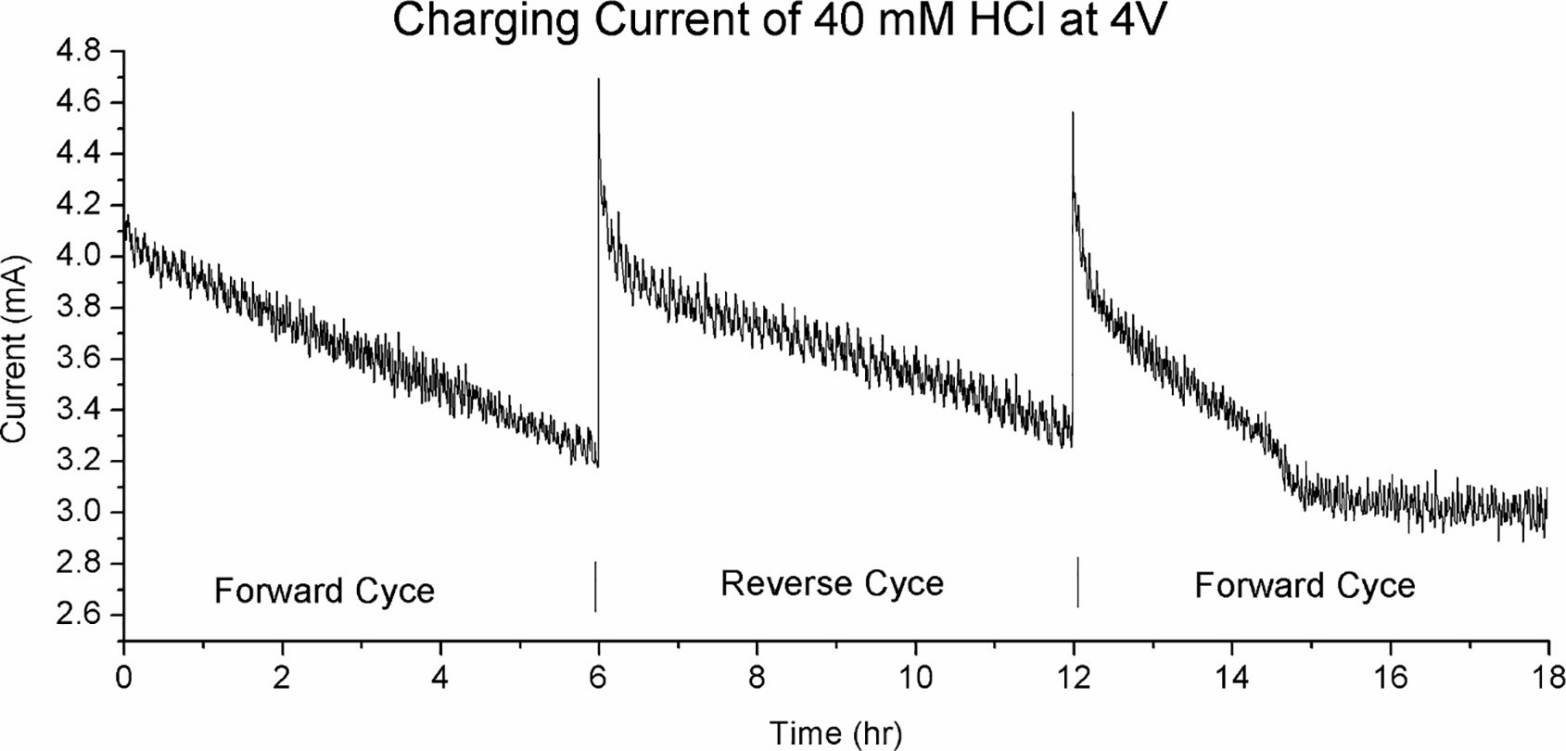
The charging current of 40 mM HCl at 4V over 3 cycles of reversing polarity, 6 hours per cycle. Here we see decreasing current through the cycle, by almost 30%. When the charging polarity is reversed the current return to near the original level before coming down slowly again.

Fig 9 shows the charging current of 40 mM HCl at 4V over 3 cycles of alternating charging voltage, 6 hours per cycle. This is from the same experiment whose charge zone profile is presented in Fig 5C. Here we see an almost linear reduction in charging current with time, by almost 30%. This is in contrast to the increasing current show in Fig 8. Again, when the polarity is reversed the current returns to the original starting level.

Decreasing current with time is what we would expect for a passive electrical system. An energy storage component (e.g. capacitor, battery) will show an initial spike and the current will decrease over time. A resistive component will generally show increase in resistance over time due to thermal heating.

The slow increase in current over a span of hours as shown in Fig 8 is unexpected. Here we observed a strong correlation between charge zone formation and charging current. The peak current is reached at around t = 180 min. This is the point that the charge zones formation reaches its peak, as shown in Fig 4. At t = 180 min when the current begins to decrease we can see a corresponding degrading of the positive zone to a lower voltage level.

In our experiments with different solutes, ions concentration and charging voltage, we can almost always see a corresponding change in the charge zones structure associated with a significant change in the charging current.

**Fig 10:**
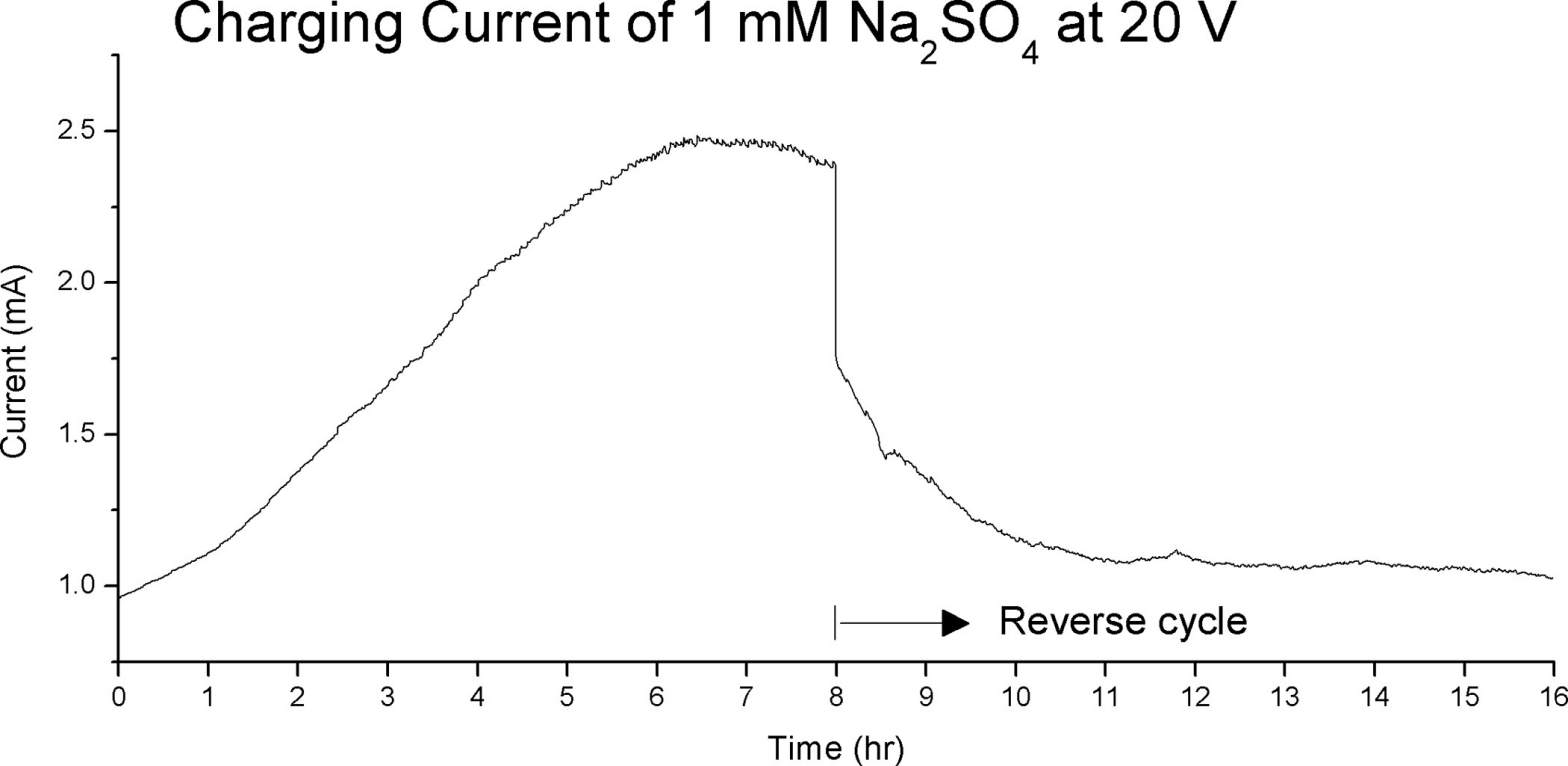
The charging current of 1 mM Na_2_SO_4_ at 20 V. The graph shows a remarkable increase of 250% over an 8 hour charging cycle. On the reverse cycle the current decay slowly back to near the original level and did not rise again.

Fig 10 show a dramatic example of the effect of charge zone formation on charging current. The solution is 1 mM Na_2_SO_4_ charging at 20 V, whose charge zone profile is shown in Fig 5A. The current rose from 1 mA to a peak 2.5 mA at t = 6.5hr, a remarkable 250% increase. At the reverse cycle we see an initial slow decay of the current which corresponds to dissipation of the charge zone formed in the previous cycle. However, new charge zones failed to form and the current stayed flat after that. Large-scale charge zone formation requires a minimum ions concentration. 1 mM ionic concentration for Na_2_SO_4_ is near this threshold.

Charge zone formation is a highly dynamic and complex phenomenon that is affected by many factors, including the charging voltage, ionic solute, ions concentration and even the hydrophilic/hydrophobic properties of the vessel. Due to space constraints, we can only present a summary of the main findings here. More detailed presentations will be the subject of follow up papers.

It is not clear at this stage the mechanisms involved that could cause 250% increase in conductivity. One possibility is that the surfaces of the charge zones can be electrically active. We have seen that the charge zones, especially the negative zone, are relatively inert and they can retain their charges for extended length of time. This means the negative zone could act as a charged body that attract a high concentration of positive ions on its surface, creating an active surface for the spread of electrons to react with the positive ions. We have seen evidence (not presented in this paper) that lead us to this conjecture.

Another possibility is that electrons could flow inside the charge zones. The negative zone especially holds a large concentration of excess electrons and it is possible that the zone could act as an N-type semiconductor for electron to flow through.

In both the above scenarios, electrons diffuse from the cathode into the water body. This means that some amount of H_2_ gas are produced away from the electrodes when the electrons meet up with the positive zone and neutralize the H^+^ ions. Another possible scenario is that the positive zone can act as a P-type semi-conductor, making the whole water body a semi-conductor medium. This is one subject of our ongoing investigations.

## Charge zones formation, ionic solute and conductivity

In this section we investigate the electrochemical properties of water with extremely low amount of ionic solutes and explore the role ionic solutes play in conductivity. We use deionized water in this experiment, which can be regarded as a solution containing minute amount of contaminants, including dissolved gas (e.g. CO_2_).

**Fig 11:**
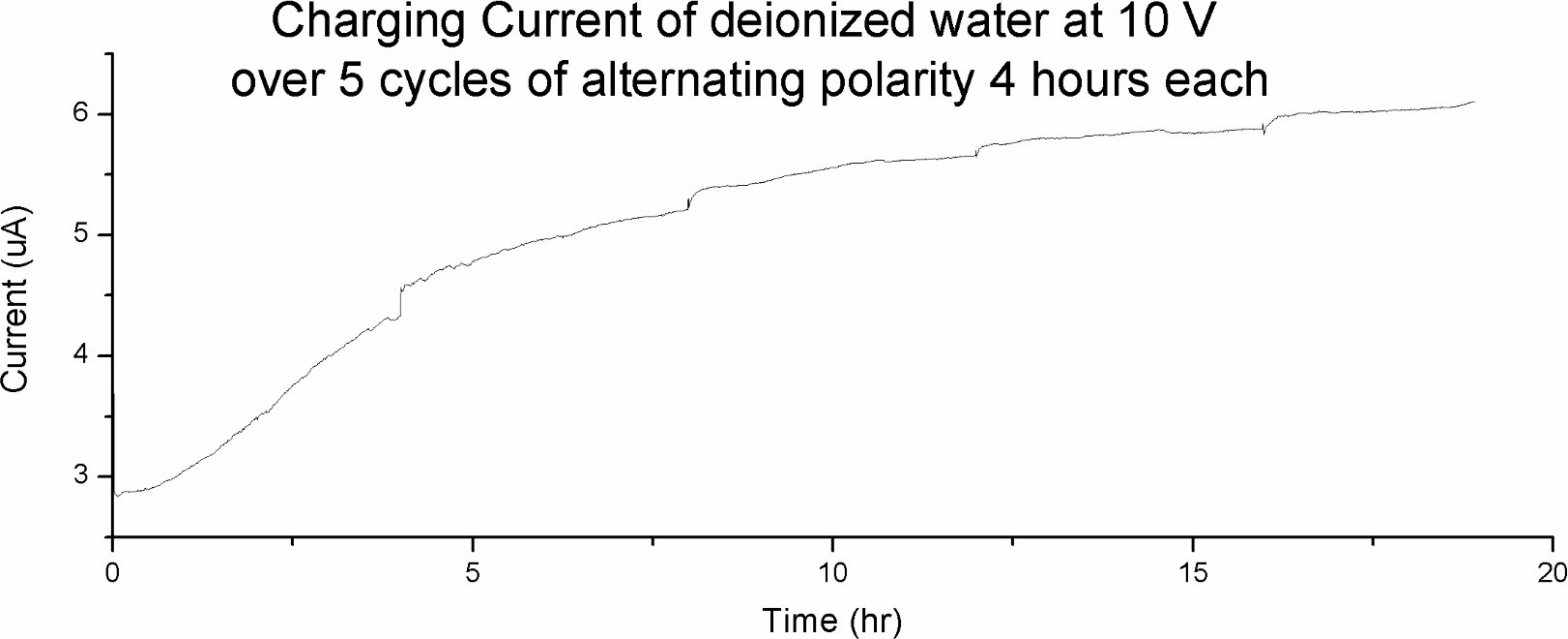
The charging current of deionized water at 10 V. The graph shows 5 cycles of alternating polarity of 4 hours each. The current builds up to 6 μA over 20 hours.

Fig 11 shows the charging current at 10 V over 5 cycles of alternating polarity of 4 hours each. The current built up to 6 μA over the 20 hours period. This is three orders of magnitude lower than the preceding experiments presented in this paper, which is what we would expect.

**Fig 12:**
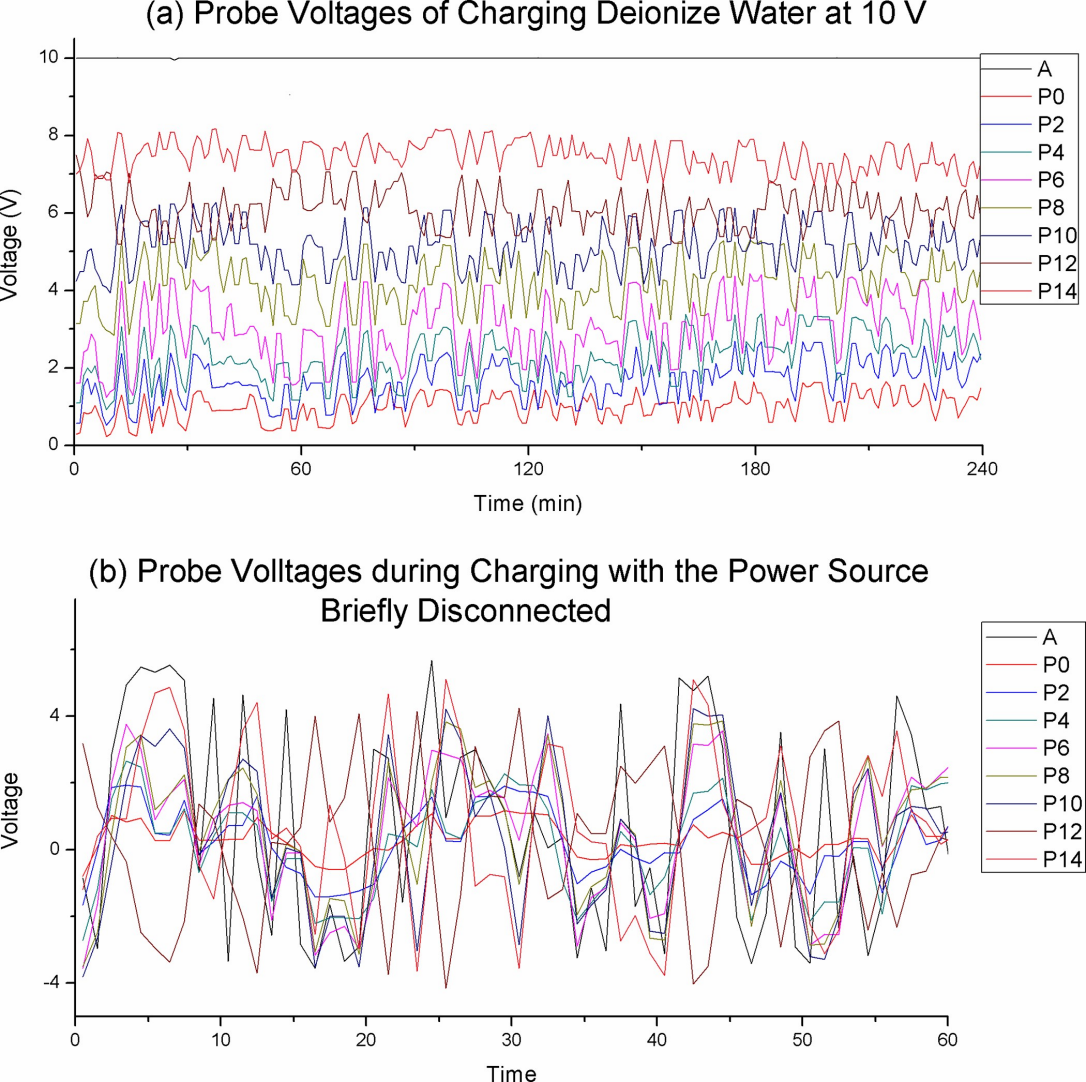
Charging of deionized water at 10 V. (a) Probe voltages. (b) Probe voltages with the power source disconnected briefly. We expected a flat line due to the lack of ionic solutes and the large random fluctuation is surprising. The ±4 V swing indicates that substantial transient charged elements of both polarity are present at both electrodes.

More interesting is the probe voltages, as shown in Fig 12. The rapid fluctuation between the -4 V to +5 V range is very surprising. We were expecting flat graphs due to the lack of ionic solutes and extremely low current. The large and rapid variation in voltage, even with an external voltage source, is indicating the present of transient charge zones at or near the electrodes.

We conjecture that charged fragments are produced at the electrodes. The anode would produce fragments containing excess positive charges while the cathode would produce fragments containing excess negative charges. Charged fragments produced at one electrode would detach and travel to the opposite electrode intermittently. The sporadic departure and arrival of charge fragments of opposing polarity at the electrodes would explain the large fluctuation measured between the electrodes.

The lacking in large-scale charge zone indicates that charge zone formation requires the presence of ionic solute. The relationship between ionic solute, charge zone formation and conductivity will be discussed further in the next section.

## Discussion–a new model of electrolysis and conductivity in water

In this paper, we demonstrated that ionic solution form large-scale charge zones in an electrolysis setup (Figs 3 and 4). Zone bearing positive voltage form on the anode while zone bearing negative voltage form on the cathode. The two charge zones expand and grow towards each other in a slow process measured in hours. We observed charge zones formation on more than 200 experiments involving a wide range of solutes, ions concentration and charging voltage and presented some representative examples in Fig 5A–5D. In Figs 6 and 7 we demonstrate that the charge zones are highly stable and have discrete voltage levels.

In Figs 8–10 we demonstrate that charge zone formation is associated with dramatic change in conductivity, both positively and negatively, and by as much as 250%. The strong relationship between charge zones is further demonstrated by our experiment with deionized water (Figs 11 and 12). No large-scale charge zone was observed in this experiment, which is expected due to the lacking in ionic solutes. But the surprisingly large and rapid fluctuation in voltage at the electrode indicate the rapid exchange of charge zone fragments. Our experiments lend overwhelming evidence that conductivity and charge zone formation is inseparable. In this section, we explore the relationship between charge zone formation and conductivity and present a new model of conductivity in ionic solution.

The common textbook description on how ionic solutions conduct electricity is a brief statement about the movement of ions—the cations are attracted to the cathode and the anions to the anode (Bard & Faulkner, 2001)-[15] but migration of ions is a one way process that does not sustain a continuous current flow. The use of AC power source, which is prevalent in the current electrochemical analysis techniques [14], would apparently avoid the ions migration problem, but it does not account for the redox reactions and their byproducts.

A more plausible mechanism is suggested by the half reactions of electrolysis, as given by:

Oxidation at anode: 2H_2_O(l) → O_2_(g) + 4H^+^(aq) + 4e^−^

Reduction at cathode: 2H^+^(aq) + 2e^−^ → H_2_(g)

The equations suggest that stream of H^+^ ions are produced at the anode and they are converted to H_2_ gas at the cathode. This is a plausible mechanism that could sustain a continuous flow of electricity, although not presented as such.

The experiments presented in this paper suggest a much more complex conductivity model as represented by the following, using NaCl solution as illustration:

- Ions migration: Cl^-^ ions migrate to the anode, and Na^+^ ions migrate to the cathode.
- At the anode O_2_ gas is released creating a concentration of H^+^ in the water. The H^+^ and Cl^-^ combine with water dipoles to form a large-scale structure. The resulting structure has an excess of H^+^ and a measurable net positive potential; it will show up as an acidic zone with pH indicators.
- Similarly at the cathode H_2_ gas is released creating a concentration of OH^-^ in the water. The OH^-^ and Na^+^ ions combine with water dipoles to form a large-scale structure. The resulting structure has an excess of OH^-^ and a measurable negative potential; it will show up as an alkaline zone with pH indicators. We have seen evidence that this structure also hold a concentration of free electrons.
- The two zones meet and neutralization takes place at the interface. Water is produced while Na^+^ and Cl^-^ are released. The ions released are carried back to the electrodes by fluid convection induced by different forces, including electrical attraction from the electrodes, gravity and electrical forces asserted by the charge zones. The cycle is complete with this.

Our model for electrolysis can be represented by the half equations below:

Oxidation at anode: 2H_2_O(l) → O_2_(g) + 4H^+^(aq) + 4e^−^

Reduction at cathode: 2H_2_O(l) + 2e^−^ → H_2_(g) + 2OH^-^(aq)

And to complete the cycle:

Neutralization at water body: H^+^(aq) + OH^-^(aq) → H_2_O(l)

The role that ionic solute play is in the formation of large scale structures that create a bridge for the H^+^ produced at the anode to neutralize the OH^-^ produced at the cathode.

We also conjecture that ionic solutes play an active role in the redox reactions. For example, a Cl^-^ ion could form unstable binding to a H2O molecule to become an ionic compound HClO^-^. The binding makes it easier for the unstable compound to give up an electron and release the oxygen atom. The Cl^-^ ions reduce the redox potential of H_2_O and acts as a catalyst in effect. Similar effect could happen at the cathode with the positive ions. The ease at which an ion can form bindings with water dipoles would provide an explanation as to why different ions display different electrolytic strength and capacity to form large-scale charge zone.

The investigations presented in this paper suggest that water’s ability to form large-scale stable structures in the presence of ionic solutes is ubiquitous. Evidence of this abounds, especially in relation to trying to mix solutions with different solutes. For example when water is added to concentrated NaOH solution the two liquid will not mix without some amount of rigorous stirring. Another common phenomenon is that water from two rivers merging resist mixing for extended length of time.

We have demonstrated that water molecules are neither random and nor inert, although its behavior is rather complex and more unpredictable than our current understanding. The current electrochemistry and electroanalytical chemistry has used various techniques to nullify the transient effect of water, primarily using AC power source and often with high degree of stirring (Bard & Faulkner, 2001) [15]. We believe that a re-evaluation of the field to include water as an active agent will yield many new insights.

Evidence of present works supports our hypothesis that the capacity for water to form stable large-scale stable structures is a key factor behind its myriad of anomalies. We conjecture that the enormous charge separation and storage capacity associated with water structuring will have a significant effect in determining the redox potential of various substances and makes water a key medium and driver for electrochemical reactions. Similarly, in biological systems water may also act as the medium for the storage and transfer of activation energy that is required by the different biological processes. Just as Chai et al. has shown that the EZ is able to transduce radiant energy to build and maintain its structure [8], we conjecture that different structural formation with different solutes may have different radiant energy absorption profiles and energy transducing characteristics. The energy mechanics that drives many of water’s anomalies would be an interesting subject for further research.

## Supporting information

S1 File **OriginGraphs**.opj Origin 8 project file that contains the experimental data for Figs 3–12.
(OPJ)

S2 File **Table 1.xsls** Excel worksheet that contains the experimental data and calculations for Table 1.
(XLSX)
